# Design and simulation of a controller system for metabolic shift regulation in mammalian cells

**DOI:** 10.1186/1753-6561-5-S8-P11

**Published:** 2011-11-22

**Authors:** Damián Baeza, Ziomara P Gerdtzen, Cristian J Salgado

**Affiliations:** 1 Laboratory for Process Modeling and Distributed Computing, Department of Chemical Engineering and Biotechnology, Faculty of Physical and Mathematical Sciences, University of Chile, Santiago, Chile; 2 Millennium Institute for Cell Dynamics and Biotechnology, Department of Chemical Engineering and Biotechnology, Faculty of Physical and Mathematical Sciences University of Chile, Santiago, Chile; 3 Department of Chemical Engineering and Biotechnology, Faculty of Physical and Mathematical Sciences, University of Chile, Santiago, Chile; 4 Centre for Biochemical Engineering and Biotechnology, Department of Chemical Engineering and Biotechnology, Faculty of Physical and Mathematical Sciences University of Chile, Santiago, Chile

## Background

Experimental evidence shows the existence of multiple steady states in mammalian cell culture with distinct cellular metabolism [1]. Different metabolic states are represented by different lactate to glucose stoichiometric ratios (ΔL/ΔG). As it is shown in Table 1, the existence of multiple steady states involves the interaction between metabolic and gene [2].

**Table 1:** Gene level fold changes corresponding to low over high ΔL/ΔG states

Enzyme	Real Time PCR	Microarray cDNA
Lactate Dehydrogenase	↓ 2.4	↓ 1.9
Pyruvate Kinase	↓ 2.9	↓ 2.0
Phosphofructokinase	↓ 2.0	↓ 1.3

Changes on the cell culture’s metabolic state have been found to be related to the amount of residual glucose in a reactor [3]. Experimental results indicate that cells in a low metabolic state respond immediately to pulse additions of glucose.

The problem of providing an optimized strategy for glucose feeding in order to achieve a specific metabolic state is yet to be studied. We propose a model based strategy for designing a control system for metabolic state regulation that considers the biological complexity of the regulation of the cellular system.

## Materials and methods

A detailed metabolic model for mammalian cell metabolism was formulated (complete model); a system of ordinary differential equations for the main metabolic variables of the following form:

wherein is the metabolite concentration vector, **S** is the stoichiometric matrix, is the reaction rate vector, and  the transport/consumption rate vector. The reaction rates can be described by:

where  is the reaction rate constant *i* and , is a non-linear function that describes the reaction-reactant dependency. Furthermore, cell growth rate was modeled as the following first order monod function:

The model’s parameters were obtained from literature and through a fitting process to experimental data; said fitting process was by the least squares method using the Nelder-Mead simplex method [4].

Once the experimental curves were obtained with the detailed model a simplified model was traced that includes the main metabolic reactions in CHO cells, and the same fitting process was carried out. Stability analysis was carried out on both the detailed and the simplified model in order to establish the number of feasible steady states for both models.

Enzyme kinetic for lactate dehydrogenase was weighted with an enzyme factor *F*, which varies between 0 and 1:

The objective of the same was to associate low *ΔL/ΔG* with low values of *F*. Based on the estimated *F* values, a Hill activation function that depends on the residual glucose concentration was adjusted to obtain the experimental curves of the culture with metabolic shift.

## Results

**Figure 1:**
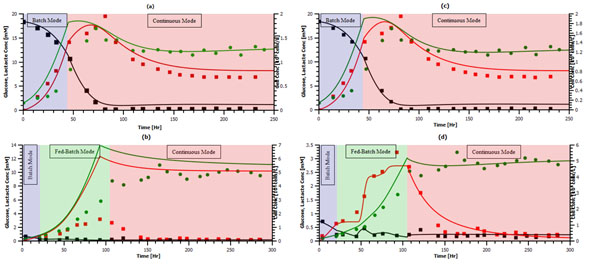
Simulation results for detailed and simplified model

The results of the curve fitting of the detailed and simplified models are depicted in Figure 1a and 1b, respectively. However, the same parameters cannot be used to simulated a cell culture that presents metabolic shift; the results of the use of the same parameters to simulate a MAK cell culture induced to a lower metabolic state do not portray the metabolic response expected for said cell culture (see Figure 1c).

Stability analysis confirmed that both the simplified and detailed metabolic models exhibit only one steady state. The existence of only one stable attractor supports the idea that metabolic regulation alone cannot explain the metabolic shift. Therefore, gene regulation is an element that should be considered in a model that correctly describes this phenomenon.

(a) Simulation results for complete model without metabolic shift, (b) Simulation results for unregulated simplified model without metabolic shift, (c) Simulation results for unregulated simplified model with metabolic shift, (d) Simulation results for regulated simplified model with metabolic shift.  : Simulated Lactate, **-** : Simulated Glucose,  : Simulated Cell Conc.,  : Lactate, ■ : Glucose,  : Cell Conc.

The implemented regulation model consists of a Hill activation function that depends on the residual glucose concentration, which modifies the enzyme kinetic for lactate dehydrogenase in the following manner:

wherein *β* = 1 is the maximal expression level, *K_glc_* = 1.24 [*mM*] is the activation coeficient, *n* = 14.64 is the Hill coeficient, and C^e^_glc_ is the glucose concentration within the bioreactor. The result of the implementation of the above function is presented in Figure 1d. The maximal expression level was fixed at 1 due to the upper limit of the named enzyme activity, the activation coefficient was retrieved from literature [3] and the Hill coefficient was adjusted through the previously used curve fitting process. Additionally, the sum of square residuals of glucose, lactate and cell concentration was 15.74 when comparing the simplified regulated model with another source of experimental data, further supporting the proposed metabolic model.

## Conclusions

Metabolic shift is caused by regulation at gene expression and metabolic levels. A reduction of the *ΔL/ΔG* ratio is accompanied by a variation in the expression levels of certain genes that participate in glycolisis, which justifies the use of a regulation function that varies from 0 to 1. Stability analysis concludes that the current metabolic models are capable of reproducing only one steady state, making explicit the need to implement a regulation model. Moreover, the immediate response capability to glucose feed pulses indicate that said regulation model must be continuous. A Hill activation function was for supplying the gene regulation due to its structure; glucose concentration has been noted experimentally to be the main factor that produces a metabolic shift in mammalian cells and the modification of enzyme kinetics leads to a different *ΔL/ΔG* ratio, thus to a different steady state.

The final objective of said model is to design a model based controller capable of maintaining a low metabolic state (ΔL/ΔG ratio under 0.5) under continuous operation. The tentative input variables are the glucose and lactate concentrations as well as the ΔL/ΔG ratio. The controller will modify the response of the system by manipulating the dilution rate and the glucose feed concentration of the culture.
